# Impregnation in the Turkey

**Published:** 1883-07

**Authors:** Edward M. Shepard


### IMPREGNATION IN THE TURKEY.

An interesting fact respecting our domestic turkey, has recently come to my notice. A friend finding that a stray turkey had come upon his premises with the intention of remaining, finally shut it up in his chicken yard, where it was permanently confined with no other associates than the chickens. The prisoner at once began to lay eggs, and, after a nest was formed, sat upon them, hatching out, in the usual time, nine healthy turkeys. Three others that had been hatched by a hen, soon died for want of care. The eggs, thirteen in all, were laid without any connection with a turkey-cock. An impregnation, then, that must have taken place before the fowl was placed in confinement, must have answered for all the eggs. Agassiz states that one copulation is supposed to answer for more than one egg in the case of the turkey, but adds that the supposition needs confirmation. The facts here mentioned seem conclusive, as there was no possible way in which connection could have taken place after the turkey was confined.

EDWARD M. SHEPARD, in "*Science.*"

